# *STK39* Polymorphism Is Associated with Essential Hypertension: A Systematic Review and Meta-Analysis

**DOI:** 10.1371/journal.pone.0059584

**Published:** 2013-03-18

**Authors:** Bo Xi, Man Chen, Giriraj R. Chandak, Yue Shen, Li Yan, Juan He, Si-Hua Mou

**Affiliations:** 1 Institute of Maternal and Child Health Care, School of Public Health, Shandong University, Jinan, People’s Republic of China; 2 School of Laboratory Medicine, Chengdu Medical College, Chengdu, People’s Republic of China; 3 Centre for Cellular and Molecular Biology, Council of Scientific and Industrial Research, Hyderabad, India; 4 Department of Epidemiology, Capital Institute of Pediatrics, Beijing, People’s Republic of China; 5 Department of Social Medicine and Health Service Management, Dalian Medical University, Dalian, People’s Republic of China; 6 Department of Pharmacy, Kunming General Hospital of Chengdu Military Commend, Kunming, People’s Republic of China; 7 Department of Clinical Laboratory, Taizhou Municipal Hospital, Taizhou, People’s Republic of China; Sanjay Gandhi Medical Institute, India

## Abstract

**Background:**

A recent genome-wide association study identified *STK39*as a candidate gene for blood pressure (BP) in Europeans. Subsequently, several studies have attempted to replicate the association across different ethnic populations. However, the results have been inconsistent.

**Objective and Methods:**

We performed a meta-analysis to elucidate the association between the *STK39* rs3754777 polymorphism (or proxy) and hypertension. Published literature from PubMed and Embase databases were retrieved and pooled odds ratio (OR) with 95% confidence interval (CI) was calculated using fixed- or random-effects model.

**Results:**

Using appropriate inclusion/exclusion criteria, we identified 10 studies that included 21, 863 hypertensive cases and 24, 480 controls from different ethnicities. The meta-analysis showed a significant association of *STK39* rs3754777 variant with hypertension (OR = 1.10, 95%CI = 1.06–1.15, *p* = 7.95×10^−6^). Further subgroup analysis by ethnicity suggested that the association was significant in Europeans (OR = 1.08, 95% CI = 1.03–1.14, *p* = 0.002) and in East Asians (OR = 1.16, 95%CI = 1.07–1.25, *p* = 4.34×10^−4^), but not in Africans (OR = 1.01, 95%CI 0.80–1.27, *p* = 0.932). We further confirmed the positive association by sensitivity analysis. No publication bias was detected (Begg’s test, *p* = 0.721; Egger’s test, *p* = 0.744).

**Conclusions:**

The present meta-analysis confirms the significant association of *STK39* polymorphism with susceptibility to hypertension in Europeans and East Asians. Future studies should include gene–gene and gene–environment interactions to investigate the identified association.

## Introduction

Hypertension, whose prevalence has increased dramatically in last few years [1], [2], is a major risk factor for cardiovascular disease and end-stage renal damage and ultimately increases mortality worldwide [3]. Hypertension is influenced by both genetic and environmental factors, as well as their interactions [4]. As per current evidence, genetic factors are estimated to account for approximately 30%–50% of variation in blood pressure (BP) levels [5].

Recently, several genome wide association studies (GWASs) have identified a host of BP/hypertension related loci in Europeans [6]–[11]. Two meta-analyses further confirmed several but not all the loci in East Asians [12], [13]. In 2009, serine/threonine kinase 39 (*STK39*) was reported as a candidate gene for BP in a GWAS on American Old Order Amish individuals [9]. In this study, a cluster of single nucleotide polymorphisms (SNPs) in the *STK39* gene were indicated to be associated with BP and two of the SNPs, rs3754777 and rs6749447 variants were replicated in an independent Amish sample, in four non-Amish Caucasian populations [9] and in a study of 1017 African Americans [14]. Since then, six publications covering 11 studies have been conducted to investigate the association between *STK39* variants and hypertension [15]–[19]. However, the results have been inconsistent.

Therefore, in this study, we performed a meta-analysis to further clarify the association between *STK39*SNP and hypertension across different ethnic populations.

## Materials and Methods

### Strategy for Literature Search

We searched the literature databases including PubMed and Embase. The search strategy to identify all possible studies involved the use of the following key words: (“Serine/threonine kinase 39” or “STK39”) and (“blood pressure” or “high blood pressure” or “hypertension”). The reference lists of retrieved articles were hand-searched. The literature search was updated on November 21, 2012.

### Inclusion Criteria and Data Extraction

The studies were included in the meta-analysis based on the following inclusion criteria: (1) evaluated the association of *STK39*SNP with hypertension; (2) used case-control or cohort design; (3) provided sufficient data for calculation of odds ratio (OR) or relative risk (RR) with 95% confidence interval (CI), e.g., provided the genotype frequencies of the studied SNP in hypertensive cases and controls. The following information was extracted from each study: (1) name of the first author; (2) year of publication; (3) country of origin;(4) ethnicity of the study population; (5)number of subjects under hypertensive cases and controls; (6) OR or RR with 95% CI; (7) diagnostic criteria for hypertensive cases and controls; (8) covariates adjustment; (9) SNPs analyzed. Two authors independently assessed the articles for compliance with the inclusion/exclusion criteria, resolved disagreements and reached a consistent decision.

### Statistical Analysis

The genotypes of studied SNP was in Hardy-Weinberg equilibrium in controls of all included studies as reported by the individual studies (*p*>0.05). We investigated the association of*STK39*SNP with hypertension by calculating pooled OR and 95% CI. The significance of pooled OR was determined by *Z* test (*p*<0.05 was considered statistically significant). Cochran’s Q test was performed to evaluate the heterogeneity between studies. A random- (DerSimonian-Laird method [20]) or fixed- (Mantel-Haenszel method [21]) effects model was used to calculate pooled OR in the presence (*p*< = 0.10) or absence (*p*>0.10) of heterogeneity, respectively. Similar approach was used for performing subgroup analysis by ethnicity. Sensitivity analysis by removing one study at a time was performed to evaluate the stability of the results. Publication bias was assessed by Begg’s test [22] and Egger’s test [23] (*p*<0.05 was considered statistically significant).All statistical analyses were performed using STATA version 11 (StataCorp LP, College Station, Texas, USA).

## Results

### Characteristics of the Studies

**Figure 1:**
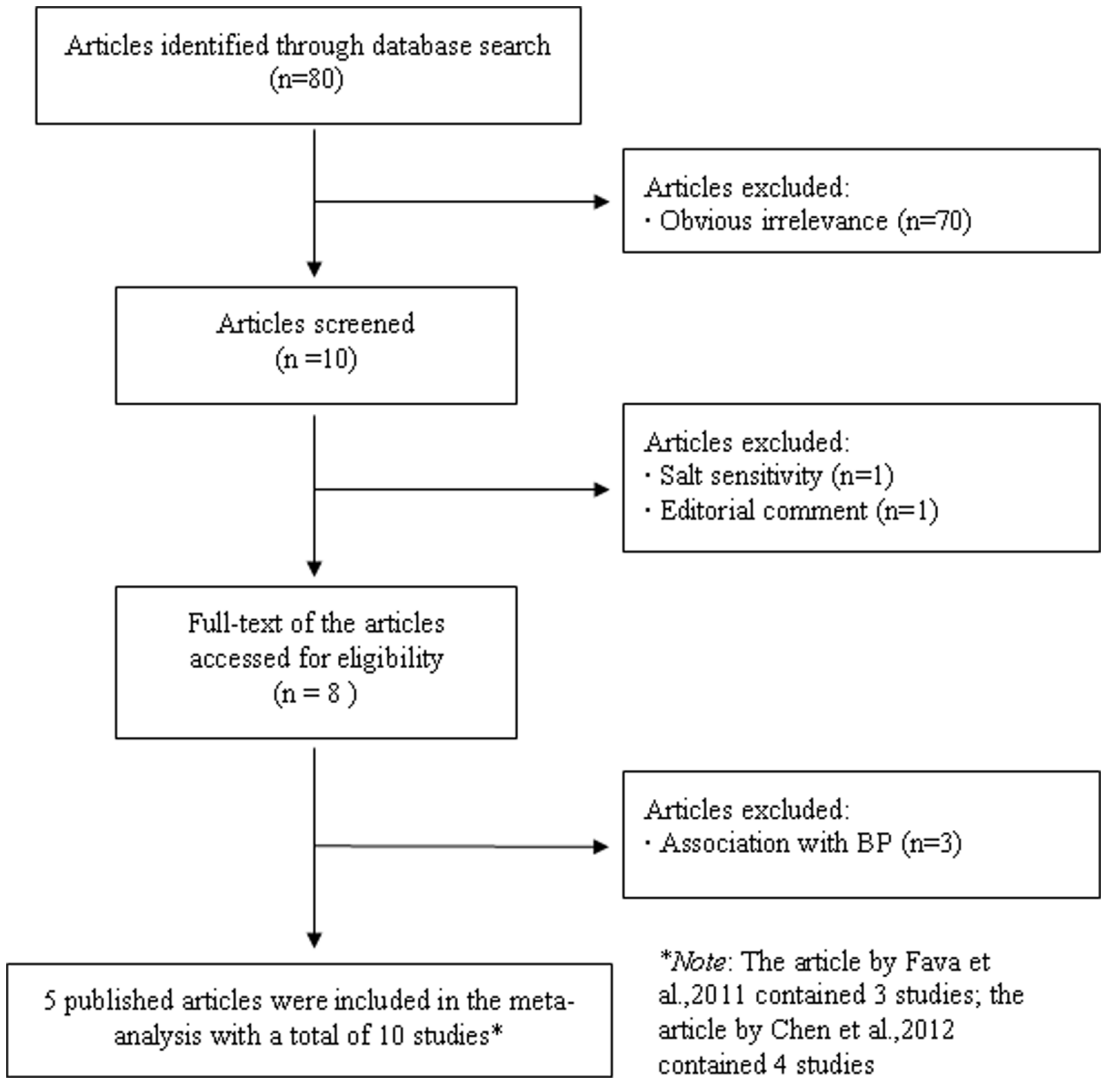
Flow chart depicting exclusion/inclusion of individual articles (or studies) for meta-analysis.

A flow chart describing the exclusion/inclusion of individual articles (or studies) has been presented as Figure 1. The comprehensive literature search identified a total of 80 potentially relevant papers, of which 70 papers were excluded because of obvious irrelevance after reading the title and/or abstract. One paper was excluded because it examined the association between *STK39*SNP and salt-sensitive hypertension [24]; another paper was excluded because it was an editorial comment [25]. Finally, 8 papers met the primary inclusion criteria, of which 3 papers were further excluded because they examined the association between*STK39* SNP and BP only [9], [14], [26]. In addition, since more than one study were included in two papers by Fava et al. [15] and Chen et al. [17], they were considered as separate studies in data analysis. Therefore, 10 studies were included in the final meta-analysis [15]–[19]. Each study had analyzed different SNP or multiple SNPs in *STK39*, however, we selected only one SNP for meta-analysis. The rs3754777 SNP is known to be in high linkage disequilibrium with rs2063958 (*r* ^2^≥0.95) and rs35929607 (*r* ^2^≥0.87). The number of cases in the included studies ranged from 266 to 6796. All the studies defined hypertension as SBP/DBP≥140/90 mmHg or currently on antihypertensive drugs. Since most studies provided covariates-adjusted OR (or RR) with 95%CI under an additive model but not the genotype frequencies of studied SNP in hypertensive cases and controls, we only calculated the pooled OR with 95%CI under an additive model. The characteristics of the included studies are listed in Table 1.

**Table 1:** Characteristics of the studies included in the meta-analysis.

Study	Country	Ethnicity	Sample size		Odds	95%	Diagnostic criteria (mmHg)		Adjustment *	Studied
			Hypertension	Controls	ratio	confidence interval	Hypertension	Controls		SNP
Fava,2011(MDC-CVA) [15]	Sweden	European	3566	2068	1.151	1.016–1.305	≥140/90^a^	<140/90	1, 2, 3, 4, 5	rs35929607
Fava,2011(MPP at baseline) [15]	Sweden	European	6028	11546	1.077	1.002–1.157	≥140/90^a^	<140/90	1, 2, 3, 4, 5	rs35929607
Fava,2011(MPP at follow-up) [15]	Sweden	European	6796	4938	1.064	0.976–1.159	≥140/90^a^	<140/90	1, 2, 3, 4,5,	rs35929607
									6, 7	
Niu, 2011 [16]	China	East Asian	548	560	1.143	0.952–1.371	≥140/90^a^	<140/90	1,2,3	rs35929607
Chen, 2012 (Population1-Male) [17]	China	East Asian	335	365	1.283	0.996–1.652	≥140/90^a^	<140/90	1, 3, 8, 9, 10	rs3754777
Chen, 2012 (Population1-Female) [17]	China	East Asian	266	244	0.906	0.667–1.230	≥140/90^a^	<140/90	1, 3, 8, 9, 10	rs3754777
Chen, 2012 (Population2-Male) [17]	China	East Asian	1330	1641	1.231	1.078–1.405	≥140/90^a^	<140/90	1, 3, 8, 9, 10	rs3754777
Chen, 2012 (Population2-Female) [17]	China	East Asian	815	812	1.092	0.909–1.313	≥140/90^a^	<140/90	1, 3, 8, 9, 10	rs3754777
Kidambi, 2012 [18]	USA	African	1155	1282	1.010	0.804–1.269	≥140/90^a^	Normal BP^b^	1, 2, 3, 11	rs2063958
Xu, 2012 [19]	China	East Asian	1024	1024	1.091	0.815–1.461	≥140/90^a^	<120/80	1, 2, 3, 9,10,	rs3754777
									12, 13, 14	

a And/or current under antihypertensive treatment.

b In the lower third of the distribution of population blood pressures.

* 1, age; 2, sex; 3, BMI; 4, heart rate; 5, interaction variables; 6, follow-up years; 7,△-BMI; 8, type 2 diabetes; 9, smoking; 10, alcohol drinking; 11, creatinine; 12,triglyceride; 13, high density lipoprotein; 14,total cholesterol.

### Meta-analysis Results

**Figure 2:**
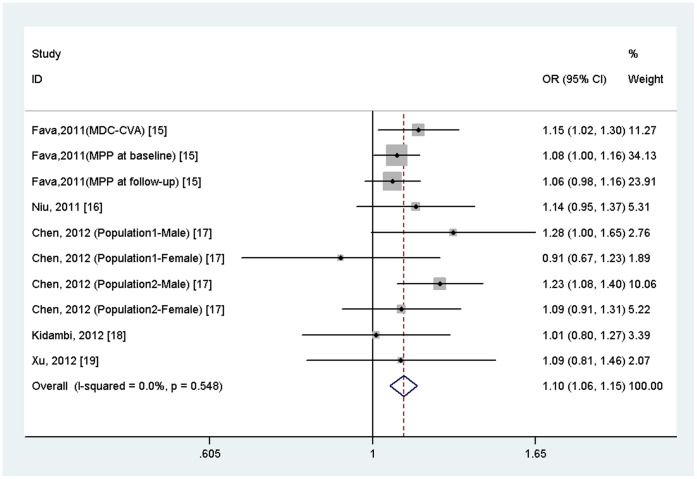
Meta-analysis of the association between *STK39* rs3754777 variant and hypertension.

**Figure 3:**
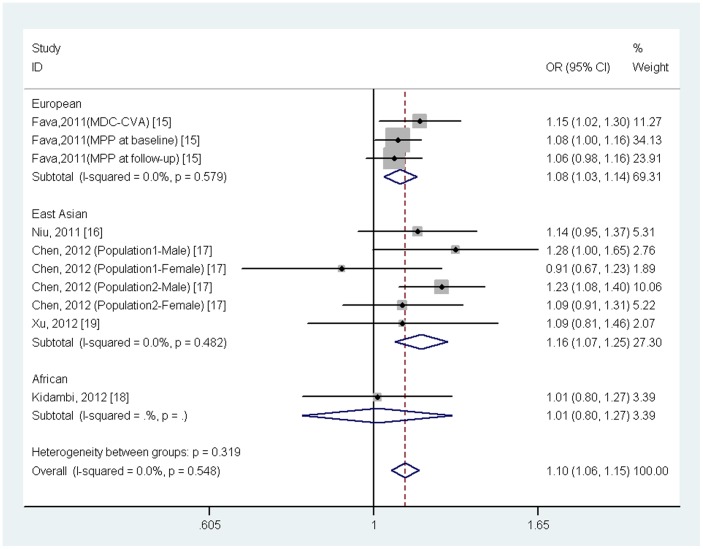
Meta-analysis of the association between *STK39*rs3754777 variant and hypertension stratified for ethnicity.

A total of 21, 863 hypertensive cases and 24, 480 controls were included in the meta-analysis. The results indicated that rs3754777 variant in *STK39* was significantly associated with hypertension (OR = 1.10, 95%CI = 1.06–1.15,*p* = 7.95×10^−6^; Figure 2), with no evidence of between-study heterogeneity (*I* ^2^ = 0.0%, *p* = 0.548). Further subgroup analysis by ethnicity showed that the association was significant in Europeans (OR = 1.08, 95% CI = 1.03–1.14, *p* = 0.002) and in East Asians (OR = 1.16, 95%CI = 1.07–1.25, *p* = 4.34×10^−4^), but not in Africans (OR = 1.01, 95%CI = 0.80–1.27, *p* = 0.932) (Figure 3).

### Sensitivity Analysis

Sensitivity analysis confirmed the significant association between rs3754777 SNP and hypertension, with OR and 95%CI ranging from 1.09 (1.04–1.14) to 1.11 (1.06–1.17). Additionally, Fava et al. [15] reported that after exclusion of 2398 subjects from the ‘Malmo Preventive Project’ (MMP) cohort, who also participated in the ‘Malmo Diet and Cancer-cardiovascular arm’ (MDC-CVA) cohort, the association between *STK39* rs35929607 and hypertension was no longer statistically significant at baseline (OR = 1.06, 95%CI = 0.98–1.14) or at follow-up (OR = 1.06, 95%CI = 0.98–1.15). We then included the above data to re-analyze the association but the overall result did not change substantially (OR = 1.09, 95%CI 1.05–1.14).

### Publication Bias

**Figure 4:**
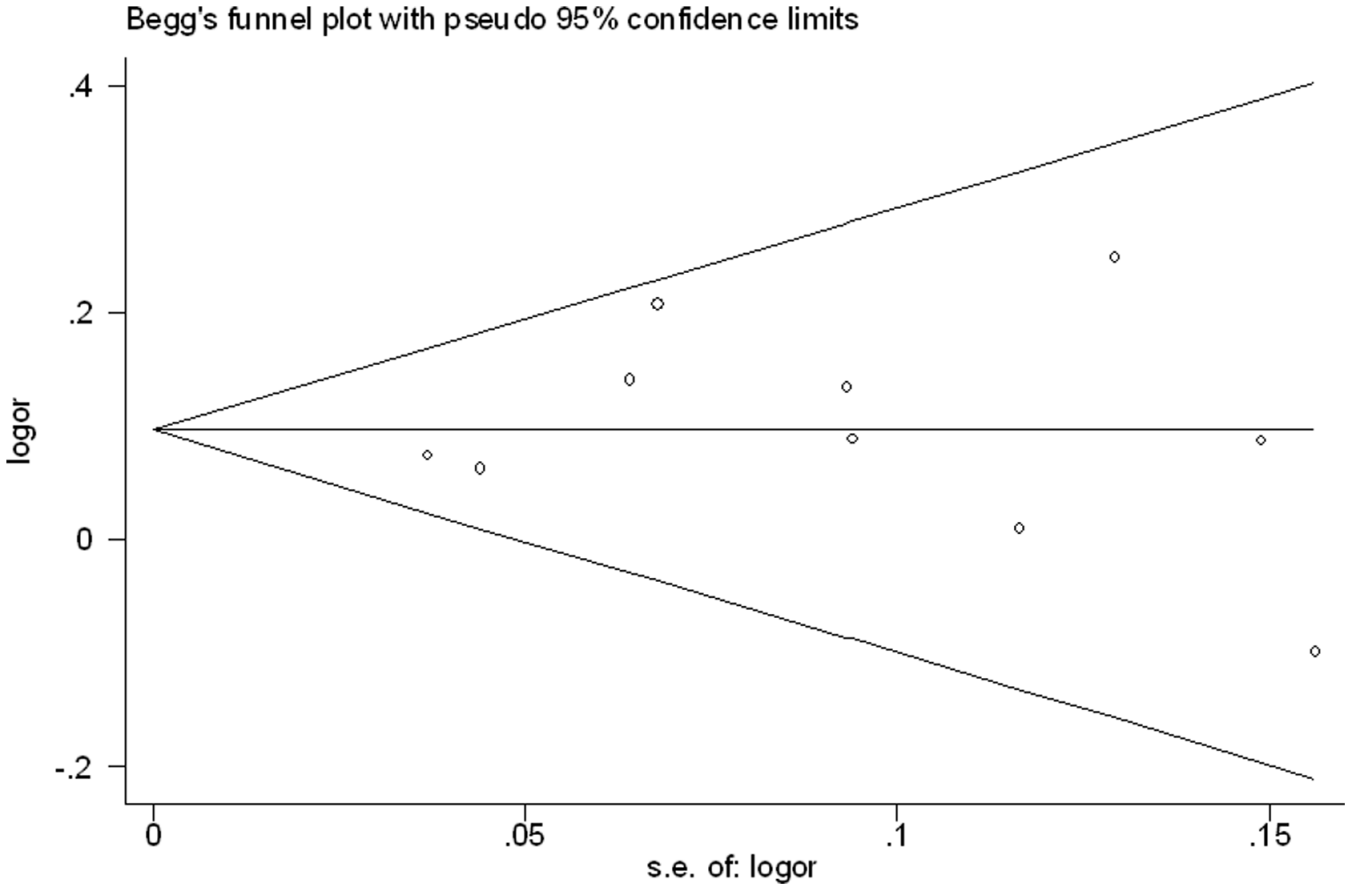
Funnel plot of the association between*STK39* rs3754777 variant and hypertension.

Begg’s funnel plot was presented as Figure 4 and no publication bias was found (Begg’s test, *p* = 0.721; Egger’s test, *p* = 0.744).

## Discussion

To our knowledge, this is the first meta-analysis assessing the association between *STK39* variants and hypertension. The result confirms that rs3754777 SNP in *STK39* is significantly associated with hypertension, and is independent of conventional environmental factors such as age, sex, body mass index (BMI) and lifestyle factors. The association was significant in Europeans and East Asians, but not in Africans. Stability of the result by sensitivity analysis and no evidence of publication bias further confirm the significant association between *STK39* rs3754777 SNP and hypertension.

The *STK39* gene encodes the Ste20-related prolinealanine-rich kinase(SPAK) protein, which may regulate BP by increasing its expression and altering renal sodium excretion through its interaction with WNK kinase and cation-chloride cotransporters [27]. Experimental studies have indicated that knock-in mice in which SPAK cannot be activated by WNKs showed significantly reduced BP that was salt-dependent [28]. Recently, two population-based studies have demonstrated that *STK39* variants might be associated with salt sensitivity [15], [24]. Thus, the above evidences support the idea that *STK39* gene is involved in salt homeostasis and thereby BP regulation in humans.

Our study has several strengths and few limitations. First, we used the OR or RR with adjustment for confounding factors from individual study to calculate the pooled OR, which increased the accuracy of effect estimate. Second, the included studies provided data on large number of subjects and greatly improved the statistical power, which made the conclusion more convincing. Third, there was no evidence of between-study heterogeneity, suggesting the homogeneity of the study populations. However, because of inability to obtain raw data, we could perform only a study-level but not a patient-level meta-analysis, which would have enabled us to adjust for multiple risk factors. This remains one of the limitations of the study. In addition, we could not address the effect of gene–gene/gene–environment interactions in this meta-analysis, though several papers have reported the effect of interaction between *STK39* SNP and sex [15], [17], BMI [19] or hormone replacement therapy [15] on susceptibility to hypertension.

In conclusion, the present meta-analysis confirmed the significant association between *STK39* variants and hypertension in Europeans and East Asians but not in the Africans. Large-scale prospective studies with consideration of gene-gene/environment interactions are required to further dissect the causal nature of identified association.
